# GOLD 2026 COPD ABE assessment tool to identify individuals at high risk in a UK multicentre COPD cohort (ERICA)

**DOI:** 10.1136/bmjresp-2025-004075

**Published:** 2026-06-25

**Authors:** Gbenga Adesoye, Alastair Watson, Tengyu Zhao, Charlotte E Bolton, Chris J Smith, Jonathan Fuld, Carmel McEniery, Joseph Cheriyan, John Cockcroft, William MacNee, Ruth Tal-Singer, Mike Polkey, Ian B Wilkinson, Marie Fisk

**Affiliations:** 1 Cambridge University Hospitals NHS Foundation Trust, Cambridge, UK; 2 University of Cambridge, Cambridge, UK; 3 Imperial College London, London, UK; 4 NIHR Nottingham BRC Respiratory Theme, University of Nottingham, Nottingham, UK; 5 Cardiff Metropolitan University, Cardiff, UK; 6 The University of Edinburgh, Edinburgh, UK; 7 Global Allergy and Airways Patient Platform, Vienna, Austria; 8 Royal Brompton Hospital, Guy's and St Thomas' NHS Foundation Trust, London, UK

**Keywords:** COPD Exacerbations, COPD epidemiology, Pulmonary Disease, Chronic Obstructive

## Abstract

Identifying people with chronic obstructive pulmonary disease (COPD) who are at high risk of significant health events such as exacerbations and hospital admissions is clinically important to guide optimisation of preventative interventions. We evaluated the utility of the Global Initiative for Obstructive Lung Disease (GOLD) 2026 ABE assessment tool which stratifies based on exacerbations or symptoms to distinguish these patients at high risk.

**Methods:**

664 participants from the ERICA (*E*valuation of the *R*ole of *I*nflammation in *C*hronic *A*irways Disease) COPD cohort were classified into GOLD ABE groups using both the Chronic Airways Assessment Tool (CAAT) score and the modified Medical Research Council (mMRC) breathlessness scale to define the degree of symptoms. National Health Service hospital episode statistics data were prospectively collected for a median follow-up of 4.75 years. Hospital admissions, including for acute exacerbations of COPD (H-AECOPD (hospitalised acute exacerbations of COPD)), were evaluated. Logistic regression analyses with ORs adjusted for potential confounding factors were performed.

**Results:**

Using CAAT, n=43/182/439 (7%/27%/66%) of participants were in groups A, B and E, respectively. By mMRC, n=144/81/439 (22%/12%/66%) were in groups A, B and E. The ABE tool derived using both CAAT and mMRC predicted H-AECOPD over follow-up. For group B versus group A by CAAT, the OR was 3.72 (95% CI 1.09 to 12.73, p=0.04), and for group E versus group A the OR was 8.13 (95% CI 2.45 to 26.92, p<0.001). By mMRC, for group B versus group A the OR was 2.69 (95% CI 1.36 to 5.32, p=0.005) and for group E versus group A the OR was 4.05 (95% CI 2.38 to 6.05, p<0.001).

**Conclusions:**

In this prospective real-world UK cohort, the simple GOLD 2026 ABE tool identified patients with high-risk COPD for severe exacerbations (H-AECOPD). The differences in groups A and B classification depending on whether the CAAT score or mMRC scale is used have clinical implications that need consideration.

**WHAT IS ALREADY KNOWN ON THIS TOPIC**

- The Global Initiative for Obstructive Lung Disease (GOLD) 2026 ABE tool is a user-friendly multidimensional tool that provides information to help categorise people with chronic obstructive pulmonary disease (COPD) according to their exacerbation risk or symptoms.

**WHAT THIS STUDY ADDS**

- This study highlights that the ABE tool predicts severe COPD exacerbations over follow-up. However, although the Chronic Airways Assessment Test (CAAT) score and modified MRC (mMRC) scale can be used interchangeably to assess symptoms in the ABE tool, they are not equivalent, and group A or B allocation may vary depending on which symptom assessment is used.

**HOW THIS STUDY MIGHT AFFECT RESEARCH, PRACTICE OR POLICY**

- It is important to highlight in COPD guidelines that the CAAT score and mMRC scale are not equivalent in assessing symptoms and may impact group allocation. As such, to undertake both symptom assessments is helpful as they provide distinct information and using the higher group allocation to guide optimisation of COPD management is a pragmatic approach.

## Introduction

 Chronic obstructive pulmonary disease (COPD) is the third leading cause of death worldwide and is a major cause of unplanned hospital admissions due to exacerbations both in the UK and globally.^1^,^4^ COPD accounts for 1 in 8 hospital admissions in the UK, is a significant cause of morbidity, and the annual economic burden of COPD on the National Health Service (NHS) in the UK is estimated to be £1.9 billion.^5 6^ COPD is a heterogeneous syndrome reflected in its different causes, endotypes and disease trajectories and association with other comorbidities.^7 8^ The ability to identify people with COPD who are at ‘high risk’ of significant health events such as exacerbations and hospital admissions due to other comorbidities is a clinically important management goal to guide treatments and optimisation of interventions. The 2026 Global Initiative for Obstructive Lung Disease (GOLD) COPD ABE assessment tool has been updated from the 2023 version. Exacerbations are now assessed separately from symptoms, reflecting the clinical priority of identifying COPD exacerbations. For individuals with no exacerbation in the preceding 12 months, symptom burden is the focus of assessment.^9 10^ As a user-friendly COPD assessment tool that does not require any specialist equipment, the ABE tool holds considerable practical promise.

To the best of our knowledge, evaluation of the GOLD 2026 COPD ABE assessment tool in a UK or global COPD cohort population has not been reported. The 2023 GOLD ABE tool has not previously been evaluated in a UK population either. Given that people with COPD often have multimorbidity and coexisting cardiovascular (CV) disease,^11 12^ it is helpful to determine whether GOLD 2026 ABE has utility in identifying people with COPD at high risk of adverse health events, besides COPD exacerbations, such as CV-related hospital admissions. Its utility will be evaluated in the context of both the GOLD grade and the BODE index (BMI (Body Mass Index), Obstruction (airflow limitation assessed by forced expiratory lung volume in 1 s (FEV_1_)), Dyspnoea (assessed by MRC score) and Exercise capacity (assessed by 6-minute walk distance (6MWD)). Both the GOLD grade and the BODE index, although they have prognostic value, require specialist equipment and input to undertake these assessments, limiting their practical utility.^13^,^16^

ERICA (*E*valuation of the *R*ole of *I*nflammation in *C*hronic *A*irways Disease) is a UK COPD cohort that is favourably positioned to evaluate the ABE 2026 assessment tool, as it is a large, prospective, longitudinal observational study containing well-characterised people with COPD with both the Chronic Airways Assessment Test (CAAT) score and the modified MRC (mMRC) scale reported. It also uniquely has baseline cross-sectional CV surrogate risk and muscle strength assessments as well as health outcomes and mortality data.^17^

The primary aim of this analysis was to evaluate the utility of the COPD 2026 ABE assessment tool to predict future severe COPD exacerbations, CV and all-cause hospitalisation and mortality in the ERICA COPD cohort. We compared the use of the CAAT or mMRC breathlessness scores to assess symptoms in the ABE tool and associations with health outcomes. We also sought to evaluate, in exploratory analysis, cross-sectional relationships of the 2026 ABE classification with baseline surrogate markers of CV risk and muscle (quadriceps) strength as unique assessments undertaken in the ERICA study. Supplementary analyses of GOLD grade and BODE index, as established prognostic classifications,^13^,^16^ were also performed to provide context for the findings.

## Methods

### Study design and study population

ERICA is a multicentre UK longitudinal observational study that evaluated the role of inflammation in COPD and COPD-associated comorbidities (namely, CV and musculoskeletal). The study and its methods have previously been described.^17^ Briefly, participants were aged ≥40 years, with physician-diagnosed COPD, a smoking history of ≥10 pack-years and post-bronchodilator FEV_1_/forced vital capacity <70% and FEV_1_<80% predicted. Participants were assessed comprehensively at baseline and linked participant data with the UK NHS electronic healthcare records (ie, Hospital Episode Statistics (HES)) and Office for National Statistics (ONS) mortality data for long-term follow-up. The median duration of follow-up was 4.75 (IQR: 4.31 to 5.28) years. Participants were recruited from 2011, and the end of follow-up was November 2017.

### Patient and public involvement

Patients and the public were not involved in the study design or results dissemination. This study involved analysis of the ERICA study database.

### Baseline assessments

Baseline assessments included self-reported exacerbations in the 12 months prior to study enrolment, the CAAT score, mMRC scale, 6-minute walk test, BMI and post-bronchodilator spirometry. Using baseline variables, the GOLD 2026 ABE assessment tool, GOLD grade (2–4) and the BODE index were computed. Baseline assessments also included arterial stiffness (augmentation index (Aix) and aortic pulse wave velocity (aPWV)) as well as carotid intima media thickness (CIMT) as surrogate CV risk markers. In addition, muscle strength was measured using the quadriceps maximal volitional contraction test (QMVC) and the short physical performance battery (SPPB) was performed. Methods for these assessments have previously been published and further information is provided in the online supplemental material.

## Outcomes of interest

### Severe COPD exacerbations

Severe COPD exacerbations (hospitalised acute exacerbations of COPD (H-AECOPD)) were identified from HES data (online supplemental table S1) and extracted from the first International Statistical Classification of Diseases and Related Health Problems, 10th Revision (ICD-10) coding position. The occurrence of H-AECOPD and time to the first H-AECOPD from baseline over longitudinal follow-up were then recorded.

### Hospitalisation

All-cause hospitalisation and CV hospitalisation were identified from HES data, and CV hospitalisation was identified using the first ICD-10 diagnosis code (online supplemental table S2 for the list of diagnoses for CV hospitalisation).

### Mortality

All-cause and cause-specific mortality for respiratory, CV, cancer or other causes were recorded in ERICA using ONS death data provided by NHS Digital and adjudicated by CV and pulmonary physicians, as previously described.^18^

### Statistical analysis

Univariate and multivariable logistic regression models were used to investigate associations of GOLD ABE, CAAT and mMRC scale at baseline with ever-occurrence during follow-up of H-AECOPD, all-cause hospitalisation, CV hospitalisation and mortality (all-cause and respiratory). Multivariable models included adjustment for age, sex, smoking status, total pack-years (TPY) and inhaled triple therapy (ITT), given baseline differences in these variables. FEV_1_ was not included in multivariable models because FEV_1_ constitutes GOLD grade and BODE index classifications. BMI was also not included in H-AECOPD, all-cause hospitalisation and mortality models because it is a component of the BODE index. For CV hospitalisation, adjustment for additional confounders that may clinically be associated with the outcome was undertaken. These included baseline history of CVD (angina and/or myocardial infarction), stroke, peripheral vascular disease, statin use, antihypertensive medication use, low-density lipoprotein/high-density lipoprotein ratio, systolic blood pressure, diabetes mellitus and BMI.

Univariate and multivariate (adjusted for age, sex, smoking status, TPY and ITT) Cox proportional hazards models were used to determine the time to the first occurrence of H-AECOPD. To assess the association between the different GOLD groups and outcomes of interest in the regression models, the lowest-risk group was the reference.

Further information is provided in the online supplemental material. Analyses involving GOLD grade (2–4) and BODE index quartiles (Q1–Q4) are reported in the online supplemental material.

Cross-sectional associations with aPWV, Aix and CIMT were adjusted for key confounding factors. For Aix, this included heart rate, age, sex and height. For aPWV, it included heart rate, mean arterial pressure, age, sex and BMI; and for CIMT, age and systolic blood pressure. QMVC was adjusted for age, sex and BMI. Bonferroni correction was undertaken for all analyses.

Summary statistics for baseline assessments are reported, and data were presented as n (%), median (IQR), mean±SD or mean±SEM for adjusted aPWV, Aix, CIMT and QMVC, unless otherwise stated. Student’s t-test, Mann-Whitney U test, Kruskal-Wallis test, χ^2^ test or one-way ANOVA, with Bonferroni corrections where appropriate, were undertaken to assess differences across groups and pairwise comparisons in baseline characteristics. A priori, a p value of 0.01 was selected as a cut-off to determine statistical significance given the number of planned analyses. Data were analysed using SPSS V.29 and Stata V.19.5, and graphs were produced using GraphPad Prism V.10.

## Results

### Baseline cohort characteristics

Within the ERICA cohort, 664 of 729 participants (91%) had complete data to enable computation of ABE using CAAT, mMRC, GOLD grade and BODE index. Table 1 shows baseline characteristics and assessments of the cohort classified by the GOLD ABE tool using the CAAT score and mMRC scale.

**Table 1:** Baseline characteristics by GOLD 2026 ABE assessment tool using CAAT score or mMRC scale

GOLD ABE							
		**GOLD CAAT**			**GOLD mMRC**		
**GOLD ABE**	**P value**	**Group A**	**Group B**	**Group E**	**Group A**	**Group B**	**P value**
n (%); total 664	–	43 (7)	182 (27)	439 (66)	144 (22)	81 (12)	–
Age (years)	0.16	71 (66–76)	68 (63–73)	67 (62–73)	69 (64–74)	67 (63–73)	0.17
Sex (M), n (%)	<0.001	32 (74)	128 (70)	247* (56)	103 (72)	57 (70)	<0.001
BMI	0.58	26.38±3.32	27.37±5.48	27.14±6.09	26.79±4.21	27.81±6.47	0.45
Current smoker, n (%)	0.009	9 (21)*	71 (39)	123 (28)	50 (35)	30 (37)	0.13
TPY	0.03	44±25	52±31	46±26	47±23	57±39	0.03
FEV_1_%	<0.001	58.77±13.46	55.30±14.98	50.02±15.98*	59.76±12.35*	49.21±16.22	<0.001
ITT, n (%)	<0.001	12 (28)*	64 (35)*	248 (57)*	36 (25)*	40 (49)*	<0.001
Inhaled LABA, n (%)	<0.001	23 (53)*	133 (73)*	383 (87)*	87 (60)*	69 (85)	<0.001
Inhaled LAMA, n (%)	<0.001	15 (35)*	104 (57)*	322 (73)*	64 (44)*	55 (68)*	<0.001
Inhaled ICS, n (%)	<0.001	22 (52)	100 (55)	333 (76)*	64 (44)*	58 (72)	<0.001
PR, n (%)	<0.001	9 (21)	50 (27)	220 (50*)	29 (20)	30 (37)	<0.001
**Comorbidities**							
CVD, n (%)	0.64	5 (12)	28 (15)	56 (13)	14 (10)	19 (23)*	0.01
AF, n (%)	0.54	2 (3)	9 (5)	31 (7)	5 (3)	6 (7)	0.28
Stroke, n (%)	0.98	3 (7)	14 (8)	33 (8)	11 (8)	6 (7)	0.99
DM, n (%)	0.20	8 (19)	19 (10)	43 (10)	15 (10)	12 (15)	0.40
PVD, n (%)	0.43	3 (7)	12 (7)	19 (4)	7 (49)	8 (10)	0.11
HTN, n (%)	0.69	20 (46)	82 (45)	184 (42)	65 (45)	37 (46)	0.69
Dyslipidaemia, n (%)	0.64	19 (44)	76 (42)	169 (38)	59 (41)	36 (44)	0.58
**CV, muscle and physical assessments**							
Aix (%)	0.97	27.07±1.16	27.10±0.55	27.24±0.34	26.55±0.63	28.05±0.84	0.35
aPWV (m/s)	0.19	10.50±0.37	10.48±0.17	10.13±0.11	10.19±0.19	11.03±0.26*	0.007
CIMT (mm)	0.07	0.84±0.03	0.87±0.01	0.83±0.01	0.86±0.02	0.87±0.02	0.08
QMVC (kg)	0.01	34.67±1.41*	30.58±0.68	30.39±0.44	32.95±0.76	28.49±1.01*	<0.001
6 MWD (m)	<0.001	450±82*	370±123*	324±130*	433±91*	302±123	<0.001
SPPB (0–12)	0.02	10.48±1.78	9.84±2.19	9.49±2.46	10.43±1.78*	9.11±2.42	<0.001
4MGS (0–4)	0.002	3.79±0.72	3.66±0.63	3.47±0.83*	3.81±0.52*	3.47±0.79	<0.001
Balance (0–4)	0.07	3.88±0.40	3.57±0.88	3.65±0.81	3.73±0.68	3.43±1.00	0.03
STS score (0–4)	0.05	2.81±1.23	2.60±1.37	2.38±1.39*	2.89±1.20	2.21±1.47	<0.001
**Outcomes**							
H-AECOPD, n (%)	<0.001	3 (7)	42 (23)	181 (41)*	19 (13)*	26 (32)	<0.001
All-cause hospitalisation, n (%)	0.05	29 (67)	124 (68)	337 (77)	99 (69)	54 (67)	0.05
CV-related hospitalisation, n (%)	0.93	6 (14)	23 (13)	53 (12)	16 (11)	13 (16)	0.53
All-cause mortality, n (%)	0.87	5 (12)	23 (13)	49 (11)	16 (11)	12 (15)	0.63
Respiratory mortality, n (%)	0.35	3 (7)	7 (4)	30 (7)	5 (4)	5 (6)	0.34

Baseline characteristics by GOLD ABE groups, classified using the CAAT or mMRC scores for symptoms.

Diagnoses included CVD (history of angina and/or myocardial infarction), AF, DM, PVD and HTN.

Aix is adjusted for age, sex, height, mean arterial pressue (MAP) and heart rate (HR); aPWV is adjusted for age, sex, BMI, MAP and HR. CIMT is adjusted for age, sex and systolic blood pressure; QMVC is adjusted for age, sex and BMI.

Adjusted values for aPWV, Aix, CIMT and QMVC are shown, as are SPPB score and scores for 4MGS and STS.

Data are presented as n (%), median (IQR), mean±SD or SEM for Aix, aPWV and CIMT.

P values are shown for across-group differences in baseline characteristics by CAAT ABE or mMRC ABE in the first and last columns, respectively.

* Pairwise comparisons between groups.

AF, atrial fibrillation; Aix, augmentation index; aPWV, aortic pulse wave velocity; BMI, Body Mass Index; CAAT, chronic airways assessment test; CIMT, carotid intima media thickness; CV, cardiovascular; CVD, cardiovascular disease; DM, diabetes mellitus; FEV_1_, forced expiratory lung volume in 1 s; GOLD, Global Initiative for Obstructive Lung Disease; H-AECOPD, hospitalised acute exacerbations of chronic obstructive pulmonary disease; HR, heart rate; HTN, hypertension; ICS, inhaled corticosteroid; ITT, inhaled triple therapy; LABA, long-acting beta agonist; LAMA, long-acting muscarinic antagonist; MAP, mean arterial pressure; 4MGS, 4-m gait speed; mMRC, modified Medical Research Council; 6MWD, 6-minute walk distance; PR, pulmonary rehabilitation (ever done); QMVC, quadriceps maximal volitional contraction test; SPPB, short physical performance battery; STS, sit-to-stand; TPY, total pack-years.

**Figure 1:**
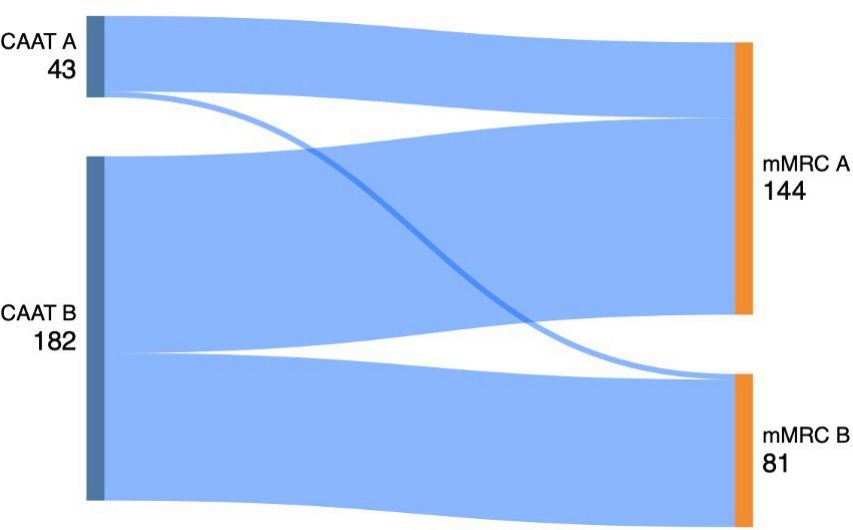
Global Initiative for Obstructive Lung Disease (GOLD) ABE tool group A or B classification by Chronic Airways Assessment Test (CAAT) score or modified Medical Research Council (mMRC) scale.

For GOLD ABE CAAT score classification, group A had the lowest number of participants (n=43; 7%), while group E had the largest (n=439; 66%). Age was similar across ABE, but group E had a lower percentage of male participants (56% vs 70%–74%, p<0.001) than group A or B. For GOLD mMRC ABE classification, there were 144 participants (22%) in group A and 81 participants (12%) in group B, while group E patients were the same with both CAAT and mMRC assessments. The trend was for the majority (57%) of group B participants defined by CAAT to be reclassified as group A (with only 3 CAAT group A participants reclassified as group B) using the mMRC scale (p<0.001; figure 1).

As expected, the distributions of FEV_1_% and ITT were significantly different across the groups for each classification, but were broadly similar when comparing the ABE classification by CAAT score and mMRC scale. Comorbidities assessed were broadly similar between the groups classified with CAAT score and mMRC, except for CVD, which was higher in group B, defined by the mMRC scale.

Baseline data for GOLD grade (2–4) and BODE are shown in online supplemental table S3. For both GOLD grade and BODE, the most severe group, GOLD grade 4 or BODE Q4, had the lowest number of participants (n=60, 9% and n=69, 11%, respectively), in contrast to GOLD ABE, where group E had the highest number of participants. The incidence of comorbidities was broadly similar between the groups of GOLD grade (2–4) and BODE index quartiles compared with GOLD ABE classification.

### Cross-sectional association with CV and muscle parameters

For GOLD ABE CAAT classification, there were no differences in Aix, aPWV or CIMT observed across groups. For ABE mMRC classification, group B had an increased aPWV versus groups A and E (group B: 11.03±0.26 m/s vs group A: 10.19±0.19 m/s and group E: 10.13±0.11 m/s, p=0.007; table 1).

For quadriceps strength evaluated by ABE CAAT score, groups B and E were weaker than group A (table 1; group B: 30.58±0.68 kg and group E: 30.39±0.44 kg vs group A: 34.67±1.41 kg, p=0.01). For mMRC, group B was the weakest (28.49±1.01 kg), and both groups B and E were weaker than group A (32.95±0.76 kg, p<0.001). For 6MWD, group E by CAAT score had the shortest distance (group E: 324±130 m vs group A: 450±82 m and group B: 370±123 m, p<0.001). For the mMRC scale, group B had the shortest distance (302±123 m), and both groups B and E had shorter distances than group A (433±91 m, p<0.001). A similar pattern was also seen for the SPPB and its composite subtests of 4-m gait speed, balance and sit-to-stand. Group E, defined by CAAT ABE, had the lowest scores (compared with groups A and B), whereas by mMRC classification, group B generally had the lowest scores (table 1). By GOLD grade and BODE index, COPD disease severity was associated with increased aPWV (6MWD as expected) and reduced QMVC and SPPB (see online supplemental material).

### Severe exacerbations (H-AECOPD) stratified using the ABE tool

**Figure 2:**
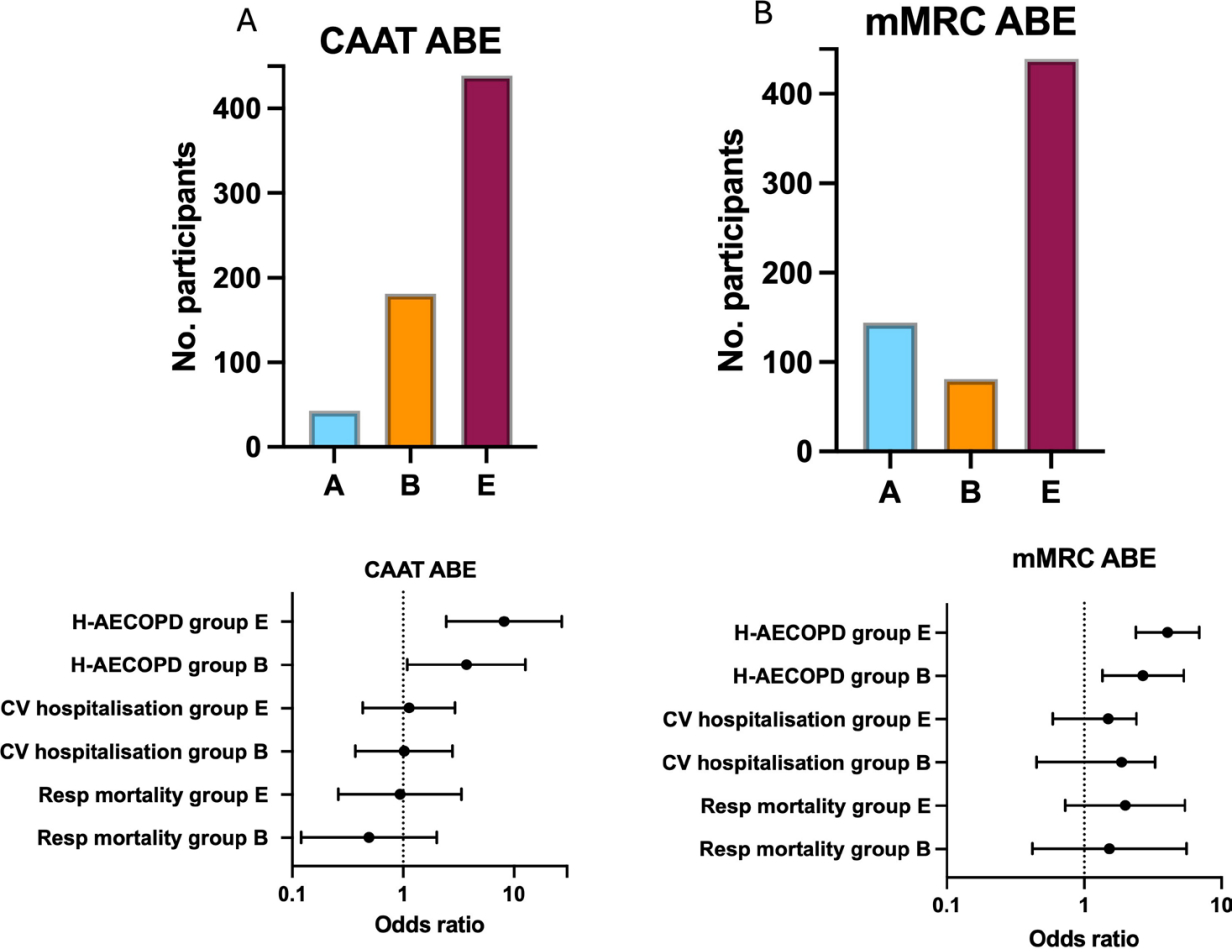
Distribution of participants and outcomes stratified by the GOLD 2026 ABE assessment tool using CAAT score or mMRC scale. CAAT, Chronic Airways Assessment Test; CV, cardiovascular; H-AECOPD, hospitalised acute exacerbations of chronic obstructive pulmonary disease; mMRC, modified Medical Research Council; No, number; resp, respiratory.

**Figure 3:**
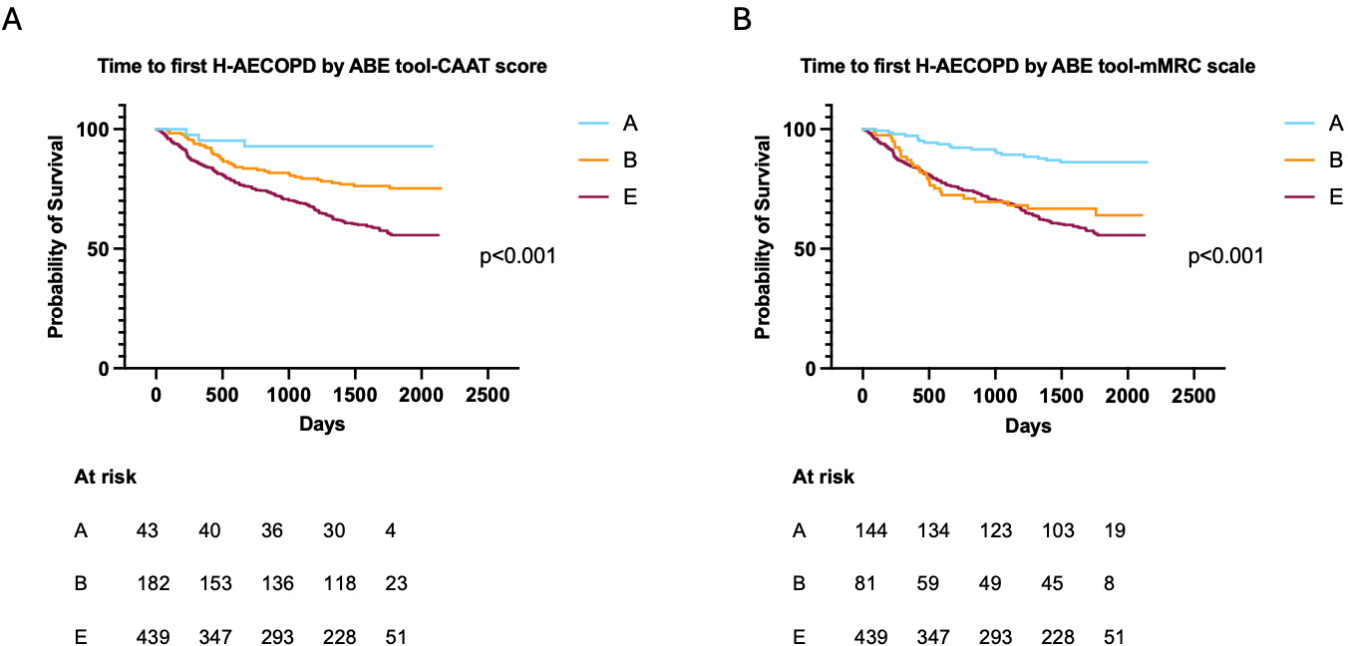
Time to the first severe exacerbation stratified by the GOLD 2026 ABE assessment tool using CAAT score or mMRC scale. CAAT, Chronic Airways Assessment Test; H-AECOPD, hospitalised acute exacerbations of chronic obstructive pulmonary disease; mMRC, modified Medical Research Council.

There was no evidence of difference in the duration of follow-up between the groups classified by the GOLD ABE assessment tool using both the CAAT score and mMRC scale (online supplemental figure S1). The number of patients with ever-occurrence of H-AECOPD was n=3/43 (7%) in group A, n=42/182 (23%) in group B and n=181/439 (41%) in group E, classified by ABE CAAT score (p<0.001). The adjusted OR for H-AECOPD was 3.72 (95% CI 1.09 to 12.73, p=0.04) for group B versus group A and 8.13 (95% CI 2.45 to 26.92, p<0.001) for group E versus group A by CAAT classification (figure 2; table 2). The Kaplan-Meier estimates of time to first H-AECOPD by groups classified with the ABE CAAT score (figure 3) were significantly different between groups (p<0.001). The adjusted HR for time to first H-AECOPD was 3.39 (95% CI 1.05 to 10.98, p=0.04) for group B versus group A and 6.22 (95% CI 1.98 to 19.59, p=0.002) for group E versus group A (table 2).

**Table 2:** Health outcomes stratified by the GOLD 2026 ABE assessment tool using CAAT score or mMRC scale

GOLD ABE	CAAT score		mMRC score	
	**Group B**	**Group E**	**Group B**	**Group E**
Severe COPD exacerbation unadjusted	4.00 (95% CI 1.18 to 13.59), p=0.003	9.35 (95% CI 2.85 to 30.70), p<0.001	3.11 (95% CI 1.59 to 6.09), p<0.001	4.62 (95% CI 2.75 to 7.75), p<0.001
Severe exacerbation adjusted	3.72 (95% CI 1.09 to 12.72), p=0.04	8.13 (95% CI 2.45 to 26.92), p<0.001	2.69 (95% CI 1.36 to 5.32), p=0.005	4.05 (95% CI 2.38 to 6.90), p<0.001
Time to first severe COPD exacerbation unadjusted	3.64 (95% CI 1.12 to 11.70), p=0.03	7.13 (95% CI 2.28 to 22.30), p<0.001	3.03 (95% CI 1.68 to 5.48), p<0.001	3.77 (95% CI 2.35 to 6.05), p<0.001
Time to first severe COPD exacerbation adjusted	3.39 (95% CI 1.05 to 10.98), p=0.04	6.22 (95% CI 1.98 to 19.59), p=0.002	2.68 (95% CI 1.47 to 4.87), p<0.001	3.36 (95% CI 2.07 to 5.44), p<0.001
All-cause hospitalisation unadjusted	1.03 (95% CI 0.51 to 2.10), p=0.93	1.60 (95% CI 0.81 to 3.13), p=0.18	0.91 (95% CI 0.51 to 1.63), p=0.75	1.50 (95% CI 0.99 to 2.27), p=0.06
All-cause hospitalisation adjusted	1.12 (95% CI 0.55 to 2.33), p=0.75	1.80 (95% CI 0.89 to 3.63), p=0.09	0.83 (95% CI 0.45 to 1.50), p=0.53	1.52 (95% CI 0.98 to 2.37), p=0.06
CV-hospitalisation unadjusted	0.89 (95% CI 0.34 to 2.35), p=0.82	0.85 (95% CI 0.34 to 2.10), p=0.72	1.53 (95% CI 0.70 to 3.37), p=0.29	1.10 (95% CI 0.61 to 1.99), p=0.76
CV-hospitalisation adjusted	1.02 (95% CI 0.348 to 3.08), p=0.97	1.28 (95% CI 0.454 to 3.61), p=0.64	1.54 (95% CI 0.62 to 3.80), p=0.35	1.50 (95% CI 0.75 to 2.99), p=0.25
All-cause mortality unadjusted	1.10 (95% CI 0.39 to 3.08), p=0.86	0.96 (95% CI 0.34 to 2.54), p=0.93	1.39 (95% CI 0.62 to 3.11), p=0.42	1.00 (95% CI 0.55 to 1.83), p=0.99
All-cause mortality adjusted	1.05 (95% CI 0.36 to 3.02), p=0.93	0.97 (95% CI 0.35 to 2.68), p=0.96	1.14 (95% CI 0.50 to 2.64), p=0.75	0.99 (95% CI 0.53 to 1.88), p=0.98
Respiratory mortality unadjusted	0.53 (95% CI 0.13 to 2.15), p=0.38	0.98 (95% CI 0.29 to 3.35), p=0.97	1.83 (95% CI 0.51 to 6.52), p=0.35	2.03 (95% CI 0.78 to 5.36), p=0.15
Respiratory mortality adjusted	0.49 (95% CI 0.12 to 2.02), p=0.32	0.94 (95% CI 0.26 to 3.35), p=0.92	1.53 (95% CI 0.42 to 5.60), p=0.52	1.99 (95% CI 0.73 to 5.42), p=0.18

Reference group: CAAT group A or mMRC group A.

ORs and HRs were unadjusted and adjusted for age, sex, smoking status, TPY and ITT. ORs for ever-occurence outcomes, HRs for time to first severe exacerbation.

CV hospitalisation was adjusted additionally for baseline CVD (myocardial infarction and/or angina), stroke, systolic blood pressure, LDL/HDL ratio, statin use, anti-HTN medication use, DM and PVD and BMI.

BMI, Body Mass Index; CAAT, chronic airways assessment test; COPD, chronic obstructive pulmonary disease; CV, cardiovascular; CVD, cardiovascular disease; DM, diabetes mellitus; GOLD, Global Initiative for Obstructive Lung Disease; HDL, high-density lipoprotein; HR, Hazard ratio; HTN, hypertension; ITT, inhaled triple therapy; LDL, low-density lipoprotein; mMRC, modified Medical Research Council; OR, Odds ratio; PVD, peripheral vascular disease; TPY, total pack-years.

Using the mMRC ABE classification, the number of patients with ever-occurrence of severe exacerbation (H-AECOPD) was n=19/144 (13%) in group A and n=26/81 (32%) in group B. This was the same for group E as per CAAT (n=181/439, 41%, p<0.001). The adjusted OR was 2.69 (95% CI 1.36 to 5.32, p=0.005) for group B versus group A, and the OR was 4.05 (95% CI 2.38 to 6.05, p<0.00) for group E versus group A (figure 2; table 2). Time to first H-AECOPD by groups classified with mMRC ABE classification is shown in figure 3 and was significantly different between the groups (p<0.001). The adjusted HR was 2.68 (95% CI 1.47 to 4.87, p=0.001) for group B versus group A, and 3.36 (95% CI 2.07 to 5.44, p<0.001) for group B versus group A (table 2).

As expected, stratification of patients by BODE index and GOLD grade predicted the occurrence of severe exacerbation and time to first exacerbation over follow-up. Results are presented in online supplementary material online supplemental Table S4.

### All-cause and CV hospitalisation stratified using the ABE tool

In group E, n=337/436 (77%) of participants had a hospitalisation over follow-up compared with 67%–69% for groups A and B, defined by the CAAT score or mMRC scale (p=0.05).

The adjusted OR for all-cause hospitalisation for group E was 1.80 (95% CI 0.89 to 3.63, p=0.09) versus group A, and for group B was 1.12 (95% CI 0.55 to 2.33, p=0.75) versus group A, using the CAAT score. For mMRC, the adjusted OR for group E versus group A was 1.52 (95% CI 0.98 to 2.37, p=0.06; table 2).

For CV hospitalisation, there was similarly no difference between groups defined by CAAT score and mMRC scale. As expected, stratification by GOLD grade and BODE index quartiles was associated with all-cause hospitalisation over follow-up (see online supplemental Table S4). There was no difference in CV hospitalisation over follow-up stratified by GOLD grade or BODE index.

### Mortality stratified by the GOLD ABE tool

GOLD ABE, defined by either CAAT or mMRC, showed no differences in all-cause or respiratory mortality across groups (table 2). BODE index and GOLD grades were associated with mortality (see online supplemental Table S4).

## Discussion

Identifying people with COPD who are at high risk of significant health events is critical for guiding optimal care and preventative interventions. To the best of our knowledge, our analysis of a large prospective observational cohort is the first to demonstrate the utility of the new GOLD 2026 COPD ABE assessment tool for predicting severe exacerbations. This has important implications for the interpretation and clinical application of this tool for tailored and optimal COPD care.

In this UK cohort of nearly 700 patients followed up over a median period of 4.75 years, the GOLD 2026^9^ ABE assessment tool differentiated between those at higher risk (groups B and E) versus lower risk group (group A) for ever-occurrence of severe (hospitalised) COPD exacerbation and time to the first severe exacerbation, when using either the CAAT score or the mMRC scale. The majority of participants (66%) in the cohort were classified as high-risk group ‘E’ based on exacerbation history, and 41% of group E had a severe exacerbation (H-AECOPD) over follow-up. There was a notable difference in classification to either group A or B, depending on which symptom instrument was used. Using CAAT, a minority (7%) were classified as group A, whereas using the mMRC scale, group B was the smallest group at 12% of the cohort. However, there were no major differences in the utility of predicting outcomes depending on whether the CAAT score or the mMRC scale was used. At baseline characterisation, however, group B, classified by the mMRC scale had several indicators of high risk. This included higher CVD (23%), increased aPWV, reduced quadriceps strength, reduced 6MWD and a lower SPPB score compared with all other groups defined by mMRC or CAAT. Crucially, the way symptom scores determine allocation to group A or B in the ABE tool has significant implications for COPD management and risk assessment. To provide context for the utility of the GOLD 2026 ABE tool, we also evaluated the GOLD grade and the BODE index. These classifications are known to be prognostic and similar to ABE, differentiating participants for severe exacerbations (H-AECOPD), but also predicting all-cause hospitalisation and all-cause and respiratory mortality over follow-up.

These data indicate that the GOLD 2026 ABE classification, which can be easily performed in any setting, even remotely or in resource-poor settings, because it is patient-reported, retains clinical utility to identify individuals who are at high risk. In the GOLD 2026 guidelines for the ABE groups (and previous GOLD 2023 ABE and 2017 iterations using ABCD)^10 19^, the CAAT score or mMRC scale can be used interchangeably for patient-reported symptoms. Although group E patients did not change based on exacerbations, the redistribution between groups A and B, depending on whether the CAAT score or mMRC scale is used within the cohort, has the potential to impact patient management, as per GOLD guidelines. The standard management recommendation for GOLD group A is a bronchodilator, whereas the group B recommendation is for a long-acting beta agonist *and* a long-acting muscarinic antagonist. This requires consideration in assessing patients in clinical practice. The authors therefore recommend that both the CAAT score and mMRC scale be used with the clinical understanding that they are not equivalent in information provided, and consider pragmatically in clinical practice whether the higher group allocation practically should guide COPD management. It is also worth emphasising the importance of additionally undertaking spirometry (GOLD grade) and multidimensional assessment, such as that provided by BODE (or at least components of it), as part of a routine work-up for patients with COPD, where feasible to implement. Especially given the additional valuable prognostic information and potential to identify individuals at higher risk from such assessments.^13^,^16^

Putting our study findings in the context of the published literature, as far as we are aware, we are one of the first studies to evaluate the GOLD 2026 ABE tool. We also examined the 2023 GOLD ABE classification in the ERICA cohort and, similarly, observed this difference between groups A and B distributions depending on whether the CAAT score or the mMRC scale was used. This issue of lack of equivalence between a CAAT score cut-off of 10 and mMRC≥2 has been noted for the 2023 ABE classification in a Spanish study of 169 patients.^20^ For the previous 2017 iteration of ABCD, this was also noted in a Korean study of ~1148 patients and also going back to the 2011 GOLD ABCD classification assessed in a UK primary care study.^21 22^ As stated above, this is important to be aware of in clinical practice for COPD management and risk assessment.

There are a few studies that evaluated the GOLD ABE 2023 classification, and they tend to be in a population cohort or registry taken from healthcare records. Our findings are generally in keeping with these published population studies. The three large population studies that assessed 2023 ABE did so using either the mMRC scale (Denmark secondary care registry cohort)^23^ or the CAAT score (Swedish National Airway Registry and the Real World Research of Diagnosis and Treatment of COPD (RealDTC) cohort in China),^24 25^ and no study assessed both the CAAT score *and* the mMRC scale group allocation in the same participants to enable comparison. The GOLD 2017 ABCD classification has been evaluated in the UK Clinical Practice Research Datalink database using MRC scores from within 5 years of the index date of the study and exacerbation history computed from healthcare records, and showed increased COPD mortality and CV mortality in historic groups B–D versus group A.^26^ ABCD was computed based on the MRC scale, and no CAAT score data were used. The 2017 ABCD iteration was superseded by the 2023 ABE tool, which has now been updated by the 2026 GOLD ABE tool. The 2026 ABE tool clearly differentiates the risk of exacerbation versus symptoms and appropriately highlights in clinical practice the importance that every exacerbation counts, identifying patients with one exacerbation in the past year as higher risk.^27^

Evaluating CV and muscle assessments by the GOLD ABE 2026 tool was an exploratory component of this analysis. The ERICA study included extra-pulmonary assessments, and the association between lung function and arterial stiffness and muscle weakness has previously been reported.^28^,^30^ As far as we are aware, this is the first study that has evaluated both CV and muscle assessments by the GOLD ABE tool. Why the ABE tool differentiated the mMRC group B participants as higher risk, identified by increased aPWV, reduced quadriceps strength, reduced 6MWD and lower SPPB score, needs consideration. This group also had a higher proportion of CVD. The CAAT and mMRC instruments assess different aspects of health. The mMRC scale specifically defines breathlessness severity; therefore, the commonality of breathlessness to CVD and respiratory disease severity is important. Indeed, a large population study without incident CVD showed the mMRC scale predicted heart failure, myocardial infarction and mortality,^31^ and a study of 182 patients with chronic cardiorespiratory disease, inclusive of asthma and interstitial lung disease as well as COPD, showed moderate correlation with the New York Heart Association Functional Classification scale for heart failure.^32^ In contrast, the multidimensional construct of the CAAT questionnaire captures COPD impact specifically on health status and quality of life.^33^ The higher OR and HR coefficients associated with H-AECOPD over follow-up for CAAT ABE classification compared with mMRC highlight how useful the CAAT score is for predicting these COPD-specific outcomes.

We also undertook analysis by GOLD grades and BODE quartiles to provide context for understanding the clinical utility of the GOLD 2026 ABE tool using CAAT or mMRC assessment. We observed an increased aPWV in GOLD grade 4 and BODE Q4, which is in keeping with published studies, since arterial stiffness is known to be inversely associated with FEV_1_.^28 29^ Muscle weakness was also associated with increased disease severity, as previously reported.^30^ The GOLD grade and the BODE index are known to have prognostic value for hospitalisation and mortality, and this was observed in our data.^16 34 35^

### Strengths and limitations

The ERICA study uniquely assessed CV and muscle parameters of participants at baseline, as well as physical performance using the 6MWD and SPPB. The CAAT score, mMRC, spirometry, BMI and 6MWD that constitute the various severity COPD classifications of ABE, GOLD grades and BODE index were done to a high standard and standardised methodology of the ERICA research study protocol, with confirmed post-bronchodilator spirometry and diagnosis of COPD. All these analyses were completed in the same patients. The cohort also has a long duration of prospective follow-up from study entry of nearly 5 years, taken from hospital episode statistics data and is representative of five centres across the UK. Mortality records were taken from the ONS and were independently reviewed by respiratory and CV physicians to determine the classification of cause of death.

Our study also has limitations. The research cohort only included participants with COPD with GOLD grade (2–4), not GOLD 1 (FEV_1_>80%), and therefore, risk classification may vary in a cohort inclusive of mild COPD. Our study is from a cohort representative across the UK (England, Wales and Scotland), but the findings, therefore, may not be translatable to other countries with different healthcare settings, and as a research cohort, these data may not be fully representative of the general COPD population in the UK. In addition, given the low number of participants in the reference group (group A), large CIs associated with some analyses are noted. Furthermore, although baseline medications were well documented, we do not have information regarding medication changes over follow-up.

## Conclusions

In summary, in this large real-world prospective UK research cohort, we demonstrate that the GOLD 2026 ABE assessment tool offers a clinically practical approach to risk stratification in patients with COPD, in keeping with the GOLD grade and the BODE index, while offering greater ease of use, since it does not require any specialist equipment. It identifies groups B and E patients as high risk for severe COPD exacerbation occurrence and time to first severe exacerbation using both the CAAT score and the mMRC.

The differences in GOLD A versus B group allocation, depending on whether the CAAT score or mMRC scale is used, should be highlighted in the COPD guidelines. A pragmatic proposal is to employ both instruments since they each provide valuable but different information, using the higher risk ABE category to guide management decisions and optimise risk stratification. Nevertheless, this work highlights the importance of these considerations for advancing guidelines and delivering high-quality COPD care.

## Supplementary material

10.1136/bmjresp-2025-004075 online supplemental file 1

## Data Availability

Data are available upon reasonable request.
