# Supplementary material for: GOLD 2026 COPD ABE assessment tool to identify individuals at high risk in a UK multicentre COPD cohort (ERICA)
Source: BMJ Open Respir Res. 2026 Jun 25;13(1):e004075. doi: 10.1136/bmjresp-2025-004075 (PMC13311567; doi:10.1136/bmjresp-2025-004075)
Supplement: online supplemental file 1 [file bmjresp-13-1-s001.docx]

**Online Supplement for**

**The GOLD 2026 COPD ABE Assessment Tool to identify high risk individuals in a**

**UK multi-centre COPD cohort (ERICA)**

**Authors:**

Authors: Gbenga Adesoye, Alastair Watson, Tengyu Zhao, Charlotte Bolton, Chris J Smith, Jonathan Fuld, Carmel McEniery, Joseph Cheriyan, John Cockcroft, William MacNee, Ruth Tal-Singer, Michael Polkey,^,^ Ian Wilkinson, Marie Fisk

# **Supplement methods**

## **Cardiovascular assessments**

### **Arterial stiffness**

Blood pressure was recorded after 15 minutes of seated rest using a validated osillometric device (HEM 750CP; Omron Corporation, Japan). Next, radial artery waveforms were recorded using a high fidelity micromanometer (SPC-301; Millar Instruments) and SphygmoCor software (AtCor Medical, Australia), from which, augmentation index (Aix), a measure of wave reflections, was calculated.^1^

After a period of supine rest and repeat supine blood pressure measurements, a high-fidelity micromanometer (SPC-301, Millar Instruments, Houston, Texas) was used to generate sequential ECG-gated pressure waveforms of the carotid and femoral arteries, recorded by SphymoCor software (AtCor Medical, Sydney, Australia). Surface distance measurements to determine wave transit times were entered into the software to generate aPWV as described previously.^2,3^

### **Carotid Intima-Media Thickness**

High-resolution B-mode ultrasound evaluated the diameter of the intima-media layers of the left and right common carotid artery, measured 1cm from the bulb. Image analysis was performed using Vascular Tools 5 software (Medical Imaging Application PLC, Coralville, USA) and the greatest CIMT was used in analyses.

### **Quadriceps Muscle Voluntary Contraction**

QMVC force was measured using the technique described by Edwards et al.^4^ Analysis were adjusted for age, sex and body mass index (BMI).

### **Six-minute walk test**

This was performed in accordance with American Thoracic Society guidelines.^5^

### **SPPB (Short Physical Performance Battery)**

This incorporates sub-tests of sub-tests: standing balance, 4‑metre gait speed (4mGS) and 5-repetition sit-to-stand (5STS).^6^

### **Hospitalisation**

Severe COPD exacerbations (hospitalised acute exacerbation of COPD: H-AECOPD) were identified from Hospital Episodes Statistics (HES) data and extracted from the first International Statistical Classification of Diseases and Related Health Problems, 10^th^ Revision (ICD-10) ICD-10 coding position.

The following ICD-10 codes were used to defined H-AECOPD, as per previous publications.^7,8^

**Table S1:** ICD-10 codes used to define hospital admission due to COPD exacerbation

| End point | ICD-10 codes |
| --- | --- |
| J22 | Lower respiratory tract infection |
| J41 | Simple and mucopurulent chronic bronchitis |
| J41.0 | Simple chronic bronchitis |
| J41.1 | Mucopurulent chronic bronchitis |
| J41.8 | Mixed simple and mucopurulent chronic bronchitis |
| J42 | Unspecified chronic bronchitis |
| J43 | Emphysema |
| J43.1 | Panlobular emphysema |
| J43.2 | Centrilobular emphysema |
| J43.8 | Other emphysema |
| J43.9 | Emphysema, unspecified |
| J44 | Other chronic obstructive pulmonary disease |
| J44.0 | Chronic obstructive pulmonary disease with acute lower respiratory infection |
| J44.1 | Chronic obstructive pulmonary disease with acute exacerbation, unspecified |
| J44.8 | Other specified chronic obstructive pulmonary disease |

Taken in first diagnosis position coding for hospital episode.

**Table S2: ICD-10 codes used to define cardiovascular hospitalisation**

| **ICD Code** | **Disease Classification** |
| --- | --- |
| I10-15 | Hypertensive diseases |
| I20 | Angina pectoris |
| I21 | Acute Myocardial Infarction |
| 120-I25 | Ischaemic Heart diseases |
| I27 (I27.0 – I27.9) | Other pulmonary hear disease |
| I34 (I34.0 – I34.9) | Non rheumatic mitral valve dx |
| I35 (I35.0 – I35.9) | Non-rheumatic aortic valve dx |
| I36 (I36.0 – I36.9) | Non-rheumatic tricuspid valve dx |
| I37 (I37.0 – I37.9) | Pulmonary valve disorders |
| I08 (I08.0 – I08.9) | Multiple valve disease |
| I42 | Cardiomyopathy |
| I44 (I44.0 – I44.7) | AVB and LBBB |
| I47 (I47.0 – I47.1) | Re-entry ventricular arrhythmia |
| I48 | Atrial fibrillation and flutter |
| I49 (I49.0 – I49.9) | Other cardiac arrythmias |
| I50 (I50.0 – I50.9) | Heart failure |
| I60 – I69 | Cerebrovascular disease |
| I70 (170.0 – 170.9) | Atherosclerosis of aorta |
| I71 (I71.0 – I71.9) | Aortic aneurysms and dissection |
| I73 (I73.0 – I73.9) | Other peripheral vascular disease |
| G45.0 – G45.9 | Transient Cerebral Ischaemic attacks and related syndromes |

Taken in first diagnosis position coding for hospital episode.

### **Statistical analysis-further information**

An important aspect of the statistical analysis undertaken was to evaluate the utility of the classifications to predict outcomes without adjustment factors, as they would be used practically in the clinic. For this reason, body mass index (BMI) and percentage predicted forced expiratory volume in 1 second (FEV_1_ %) were not included in ABE models because these are variables in BODE index (both BMI and FEV_1_ %) and GOLD grade (FEV_1_ %). Factors selected for adjusted analysis were based on demographic factors and other factors that may be confounding factors for all outcomes assessed. These included age, sex, smoking status, total pack years and inhaled triple therapy.

In addition, for cardiovascular hospitalisation, cardiovascular comorbidity (history of myocardial infarction and/or angina), stroke, systolic blood pressure, low density lipoprotein (LDL)/ high density lipoprotein (HDL) ratio, statin use, anti-hypertension medication use , , , peripheral vascular disease (PVD), diabetes mellitus (DM) and body mass index (BMI), were included in models.

Group A was the reference group for ABE, GOLD grade 2 for GOLD grade and Q1 for the BODE Index.

# **Results**

There was no significant difference in duration of follow up for the cohort evaluated by ABE tool using either the Chronic Airways Assessment Tool (CAAT) score or modified MRC scale, Figure S1.


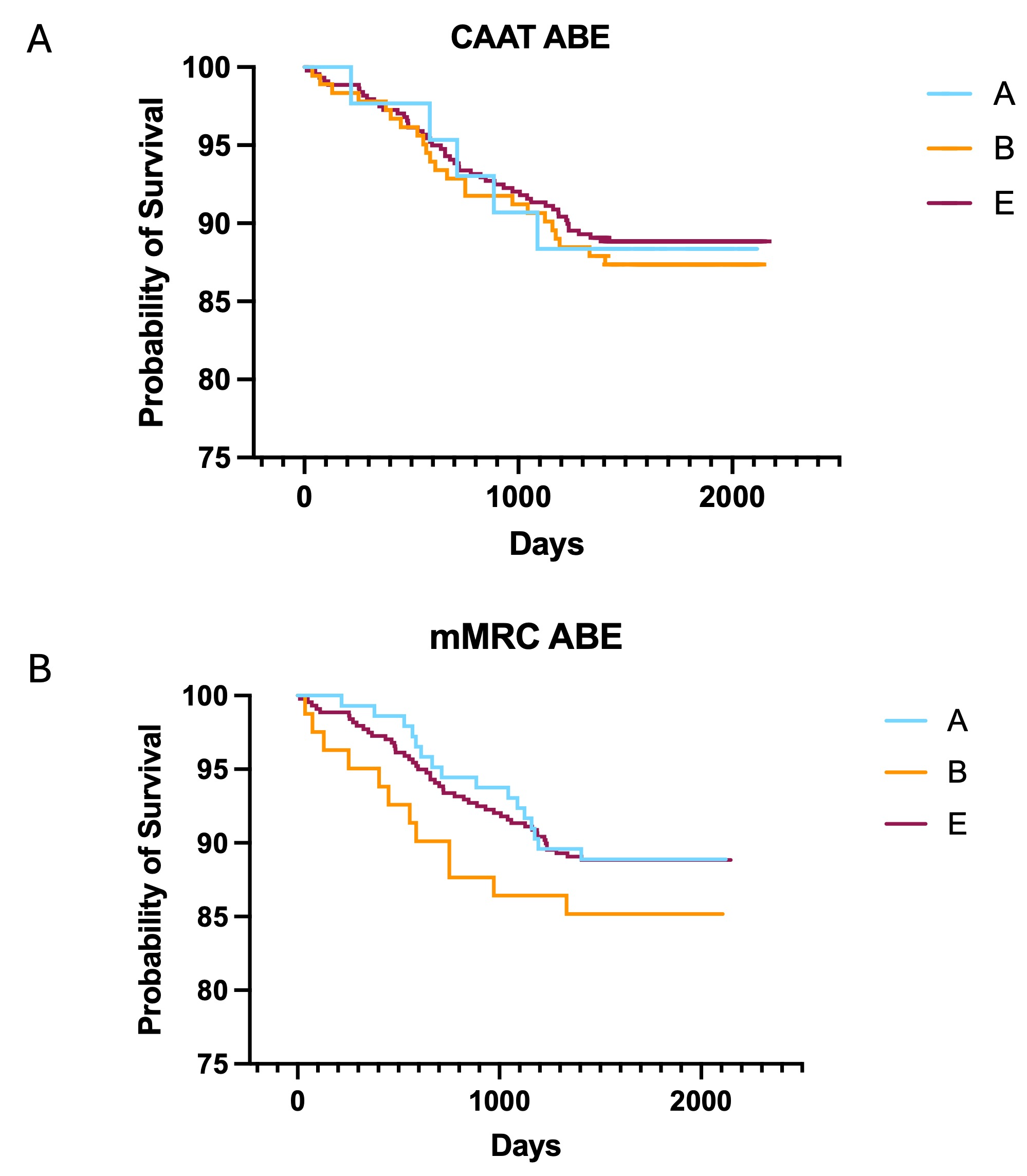
**Figure S1**: Duration of follow-up of ABE groups by CAAT score or mMRC sca

**Table S3: Cohort demographics**

|  | **GOLD Grade** | | | | **BODE Index quartiles** | | | | |
| --- | --- | --- | --- | --- | --- | --- | --- | --- | --- |
|  | **2** | **3** | **4** | **P value** | **Q1** | **Q2** | **Q3** | **Q4** | **P value** |
| N (%) | 371 (56%) | 233 (35%) | 60  (9%) | - | 307  (46%) | 159  (24%) | 129  (19%) | 69  (11%) | - |
| Age (years) | 68  [63,74] | 68  [62,73] | 65  [61,70] | 0.06 | 68  [63,72] | 67  [62,73] | 68  [62,74] | 68  [62,73] | 0.61 |
| Sex (M) | 224 (60%) | 135  (58%) | 48  (80%)* | 0.006 | 205  (67%) | 87  (55%) | 72  (56%) | 43  (62%) | 0.04 |
| BMI | 28.10±  5.39 | 26.48±  6.02 | 23.87±  5.76* | <0.001 | 27.28±  4.38 | 26.71±  5.91 | 28.33±  7.58 | 25.29±  7.58 | 0.03 |
| Current smoker | 109 (29%) | 80 (34%) | 14 (23%) | 0.19 | 84  (27%) | 58  (36%) | 39  (30%) | 22  (32%) | 0.24 |
| TPY | 47±27 | 48±28 | 47±26 | 0.69 | 45±25 | 48±32 | 47±26 | 54±28 | 0.12 |
| FEV_1_ % | 64±9% | 40±6%* | 23±4%* | <0.001 | 62±11* | 50±14 | 42±13 | 33±11* | <0.001 |
| ITT | 141 (38%)* | 145 (62%) | 38  (63%) | <0.001 | 115  (37%)* | 83  (52%) | 80  (62%) | 46  (67%) | <0.001 |
| Inhaled LABA | 275 (74%)* | 207 (89%) | 57 (95%) | <0.001 | 222 (72%)* | 138 (87%) | 114 (88%) | 65 (94%) | <0.001 |
| Inhaled LAMA | 217 (58%)* | 175 (75%) | 49 (82%) | <0.001 | 175 (57%)* | 114 (72%) | 96 (74%) | 56 (81%) | <0.001 |
| Inhaled ICS | 218 (59%)* | 187 (80%) | 50 (83%) | <0.001 | 178 (58%)* | 114 (72%) | 103 (80%) | 60 (87%) | <0.001 |
| PR | 111 (30%)* | 123 (53%) | 45  (75%) | <0.001 | 84 (27%)* | 78 (49%) | 74 (57%) | 43 (62%) | <0.001 |
| **Co-morbidities** | | | | | | | | | |
| CVD  n (%) | 83  (22%) | 38  (16%) | 8  (13%) | 0.09 | 54  (18%) | 31  (19%) | 28  (22%) | 16  (23%) | 0.63 |
| AF  n (%) | 21 (6%) | 19 (8%) | 2  (3%) | 0.29 | 17  (6%) | 8  (5%) | 10  (8%) | 7 (10%) | 0.41 |
| Stroke  n (%) | 32  (9%) | 15  (6%) | 3  (5%) | 0.46 | 23  (7%) | 11  (7%) | 8  (6%) | 8  (12%) | 0.57 |
| DM  n (%) | 39  (11%) | 24  (10%) | 7  (12%) | 0.95 | 26  (8%) | 19  (12%) | 16  (12%) | 9  (13%) | 0.45 |
| PVD  n (%) | 20  (5%) | 12  (5%) | 2  (3%) | 0.80 | 13  (4%) | 8  (5%) | 6  (5%) | 7  (10%) | 0.25 |
| HTN  n (%) | 165  (44%) | 99  (42%) | 22  (37%) | 0.51 | 135  (44%) | 69  (43%) | 56  (43%) | 26  (38%) | 0.81 |
| Dyslipidaemia (%) | 159  (43%) | 90  (39%) | 15  (25%) | 0.04 | 122  (40%) | 72  (45%) | 45  (35%) | 25  (36%) | 0.31 |
| **Cardiovascular, Muscle and physical assessments** | | | | | | | | | |
| Aix (%) | 26.88±  0.40 | 27.87±  0.51 | 26.45±  1.03 | 0.23 | 26.84±  0.45 | 27.57±  0.62 | 27.77±  0.69 | 26.76±  0.96 | 0.60 |
| aPWV (m/s) | 10.07±  0.12 | 10.29±  0.15 | 11.41±  0.33 | <0.001 | 9.91±  0.13 | 10.18±  0.18 | 10.69±  0.21 | 11.03±  0.29 | <0.001 |
| CIMT (mm) | 84±  0.01 | 84±  0.01 | 82±  0.03 | 0.59 | 0.85±  0.01 | 0.84±  0.02 | 0.83±  0.02 | 0.81±  0.02 | 0.51 |
| QMVC (kg) | 32.44±  0.47 | 29.10±  0.60 | 26.25±  1.19 | <0.001 | 33.54±  0.49 | 30.39±  0.69 | 27.72±  0.77 | 24.04±  1.07 | <0.001 |
| 6 MWD (m) | 374±  125 | 318±  131 | 266±  106 | <0.001 | 433±  76 | 336±  108 | 248±  99 | 159±  81 | <0.001 |
| SPPS (0-12) | 9.86±  2.28 | 9.39±  2.42 | 9.27±  2.50 | 0.02 | 10.67±  1.61 | 9.66±  2.22 | 8.36±  2.34 | 7.45±  2.84 | <0.001 |
| 4MGS (0-4) | 3.62±  0.75 | 3.47±  0.80 | 3.37±  0.88 | 0.02 | 3.86±  0.43 | 3.58±  0.72 | 3.12±  0.91 | 2.84±  1.04 | <0.001 |
| Balance (0-4) | 3.61±  0.85 | 3.63±  0.82 | 3.85±  0.48 | 0.10 | 3.75±  0.71 | 3.67±  0.73 | 3.51±  0.94 | 3.32±  1.05 | <0.001 |
| STS score  (0-4) | 2.64±  1.32 | 2.30±  1.42 | 2.13±  1.42 | 0.002 | 3.07±  1.10 | 2.41±  1.33 | 1.73±  1.31 | 1.36±  1.31 | <0.001 |
| **Outcomes** | | | | | | | | | |
| H-AECOPD | 76 (20%) | 108 (46%) | 42  (70%)* | <0.001 | 54 (18%)* | 61 (38%) | 64 (50%) | 47 (68%)* | <0.001 |
| All-cause  hospitalisation | 261  (70%) | 177 (76%) | 52 (87%) | 0.02 | 210 (68%)* | 118 (74%) | 100 (78%) | 62 (89%)* | 0.002 |
| CV-related hospitalisation | 40  (11%) | 34 (15%) | 8 (13%) | 0.37 | 28  (9%) | 24 (15%) | 20 (16%) | 10 (15%) | 0.14 |
| All-cause mortality | 34  (9%) | 27 (12%) | 16 (27%)* | <0.001 | 24 (8%) | 17 (11%) | 15 (12%) | 21 (30%)* | <0.001 |
| Respiratory mortality | 12  (3%) | 14 (6%_ | 14 (23%)* | <0.001 | 5  (2%)* | 10  (6%) | 11  (9%) | 14 (20%) | <0.001 |

BMI= body mass index, TPY=total pack years, FEV_1_=forced expiratory lung volume, ITT=inhaled triple therapy (LABA+LAMA+ICS). LABA= long acting beta agonist, LAMA=long acting muscarinic antagonist, ICS= inhaled corticosteroid, PR=pulmonary rehabilitation (ever done). Diagnoses of CVD=cardiovascular disease (history of angina, and/or myocardial infarction), AF=atrial fibrillation, DM=diabetes mellitus, PVD=peripheral vascular disease, HTN=hypertension. Adjusted Aix= augmentation index for age, sex, height, mean arterial pressure (MAP) and heart rate (HR), aPWV= aortic pulse wave velocity adjusted for age, sex, BMI, MAP and HR, CIMT=carotid intima-media thickness adjusted for age, sex, systolic blood pressure, QMVC=quadriceps muscle voluntary contraction adjusted for age, sex, BMI. aPWV, Aix, CIMT and QMVC adjusted values are shown. 6MWD=six-minute walk distance. SPPB=short physical performance battery score and scores for 4MGS (4-metre gait speed) and STS (sit-to-stand) shown. Data presented as n (%), median [interquartile range], mean±standard deviation or standard error of the mean for Aix, aPWV, CIMT. H-AECOPD= hospitalised acute COPD exacerbation. Significant differences across 3 groups compared using Kruskal Wallis test (for age where no significant difference), or Chi square, or 1-way ANOVA, both using Bonferroni correction, pairwise comparison shown by *. P-values shown for across group differences for baseline characteristics by GOLD grade and BODE index quartiles.

**Table S4: Health outcomes stratified by GOLD grade and BODE index quartiles.**

| **Classification** | **GOLD Grade** | | **BODE Index Quartiles** | | |
| --- | --- | --- | --- | --- | --- |
| **Outcome** | **3** | **4** | **Q2** | **Q3** | **Q4** |
| Severe COPD exacerbation  unadjusted | 3.11  (2.14, 4.81)  p<0.001 | 8.66 (4.64,16.14)  p<0.001 | 2.92 (1.89,4.50)  p<0.001 | 4.61  (2.93, 7.26) p<0.001 | 10.01  (5.57, 17.97)  p<0.001 |
| Severe COPD exacerbation adjusted | 3.36 (2.34,4.51)  p<0.001 | 9.42 (5.09,17.46)  p<0.001 | 2.80 (1.80,4.36)  p<0.001 | 4.47 (2.81,7.12)  p<0.001 | 9.24 (5.09,16.80)  p<0.001 |
| HR  Time to first severe COPD exacerbation  unadjusted | 2.71  (2.02, 3.63)  p<0.001 | 6.33  (4.33,9.26)  p<0.001 | 2.42  (1.67, 3.52)  p<0.001 | 3.59 (2.48,5.23)  p<0.001 | 6.31  (4.19,9.49)  p<0.001 |
| HR Time to first severe COPD exacerbation  adjusted | 2.70 (2.01,3.63)  p<0.001 | 6.37 (4.34,9.35)  p<0.001 | 2.55 (1.76,3.69)  p<0.001 | 3.78 (2.63,5.45)  p<0.001 | 7.00 (4.72,10.40)  p<0.001 |
| All-cause hospitalisation unadjusted | 1.33  (0.92, 1.94) p=0.13 | 2.74 (1.26,5.96) p=0.01 | 1.33 (0.87,2.04)  p=0.19 | 1.59 (0.99,2.57) p=0.06 | 4.09 (1.81,9.27)  p<0.001 |
| All-cause hospitalisation adjusted | 1.35 (0.91,2.00)  p=0.13 | 2.98 (1.35,6.06)  p=0.007 | 1.33 (0.86,2.06)  p=0.20 | 1.63 (0.99, 2.70)  p=0.06 | 3.93 (1.71, 9.00)  p=0.001 |
| CV-hospitalisation unadjusted | 1.41 (0.87,2.31)  p=0.17 | 1.27 (0.56,2.87) p=0.56 | 1.77 (0.99,3.17)  p=0.05 | 1.83 (0.99,3.88)  p=0.06 | 1.69 (0.78,3.67),  p=0.19 |
| CV-hospitalisation adjusted | 1.52  (0.87,2.65)  p=0.14 | 1.54 (0.62,3.83)  p=0.35 | 1.92 (1.02,3.64)  p=0.05 | 1.68 (0.84,3.63)  p=0.14 | 1.49(0.60,3.69  p=0.30 |
| All-cause mortality unadjusted | 1.30 (0.76,2.22)  p=0.34 | 3.60 (1.84,7.06)  p<0.001 | 1.42 (0.74,2.71)  p=0.30 | 1.55 (0.79,3.07)  p=0.21 | 5.16 (2.66,9.99)  p<0.001 |
| All-cause mortality adjusted | 1.15 (0.65,2.00)  p=0.62 | 3.70 (1.81,7.57)  p<0.001 | 1.35 (0.69,2.63)  p=0.38 | 1.45 (0.72,2.92)  p=0.31 | 4.62 (2.31,9.25)  p<0.001 |
| Respiratory mortality unadjusted | 1.91  (0.87,4.21) p=0.11 | 9.11  (3.97, 20.88)  p<0.001 | 4.05 (1.36,12.07)  p=0.01 | 5.63 (1.92,16.55)  p=0.002 | 15.38 (5.32,44.41)  p<0.001 |
| Respiratory mortality adjusted | 1.73 (0.77,3.91)  p=0.18 | 10.16 (4.15,24.81)  p<0.001 | 3.97 (1.31,11.97)  p=0.02 | 5.42 (1.81,16.22)  p=0.003 | 14.30 (4.81,42.53)  p<0.001 |

Reference group= GOLD grade 2, BODE Index Q1 (quartile 1). Odds ratio (OR) unadjusted, and OR adjusted for age, sex, smoking status, total pack years, inhaled triple therapy. Hazard ratio (HR) unadjusted and HR adjusted for age, sex, smoking status, total pack years, inhaled triple therapy. CV: Cardiovascular hospitalisation was adjusted additionally for baseline CVD (myocardial infarction and/or angina), stroke, seated systolic blood pressure, low density lipoprotein(LDL)/high density lipoprotein(HDL) ratio, statin use, anti-hypertension medication use, diabetes mellitus, peripheral vascular disease and BMI (except for BODE as BMI already included).

**References:**

1. Pauca AL, O’Rourke MF, Kon ND. Prospective evaluation of a method for estimating ascending aortic pressure from the radial artery pressure waveform. *Hypertension*. 2001;38:932–937.
2. Wilkinson IB, Fuchs SA, Jansen IM, Spratt JC, Murray GD, Cockcroft JR, Webb DJ. Reproducibility of pulse wave velocity and augmentation index measured by pulse wave analysis. *J Hypertens*. 1998;16(12 pt 2):2079–2084.
3. Fisk M, McEniery CM, Gale N, Mäki-Petäjä K, et al.. Surrogate Markers of Cardiovascular Risk and Chronic Obstructive Pulmonary Disease: A Large Case-Controlled Study. Hypertension. 2018 Mar;71(3):499-506.
4. Edwards RH, Young A, Hosking GP, et al. Human skeletal muscle function: description of tests and normal values. Clin Sci Mol Med 1977;52:283–90.
5. ATS Committee on Proficiency Standards for Clinical Pulmonary Function Laboratories. ATS statement: guidelines for the six-minute walk test. Am J Respir Crit Care Med. 2002 Jul 1;166(1):111-7. doi: 10.1164/ajrccm.166.1.at110
6. Mohan D, Benson VS, Allinder M, Galwey N, Bolton CE, Cockcroft JR, MacNee W, Wilkinson IB, Tal-Singer R, Polkey MI; ERICA Consortium. Short Physical Performance Battery: What Does Each Sub-Test Measure in Patients with Chronic Obstructive Pulmonary Disease? Chronic Obstr Pulm Dis. 2020 Jan;7(1):13-25. doi: 10.15326/jcopdf.7.1.2019.0144. PMID: 31999899; PMCID: PMC7182385
7. Fermont JM, Bolton CE, Fisk M, Mohan D, Macnee W, Cockcroft JR, McEniery C, Fuld J, Cheriyan J, Tal-Singer R, Wilkinson IB, Wood AM, Polkey MI, Müllerova H. Risk assessment for hospital admission in patients with COPD; a multi-centre UK prospective observational study. PLoS One. 2020 Feb 10;15(2):e0228940.
8. Pavey H, Polkey MI, Bolton CE, Cheriyan J, McEniery CM, Wilkinson I, Mohan D, Casaburi R, Miller BE, Tal-Singer R, Fisk M. Circulating testosterone levels and health outcomes in chronic obstructive pulmonary disease: results from ECLIPSE and ERICA. BMJ Open Respir Res. 2023 Jun;10(1):e001601.
